# Clinical and Pharmacological Aspects of Inflammatory Demyelinating Diseases in Childhood: An Update

**DOI:** 10.2174/157015910791233141

**Published:** 2010-06

**Authors:** Alberto Spalice, Pasquale Parisi, Laura Papetti, Francesco Nicita, Fabiana Ursitti, Francesca Del Balzo, Enrico Properzi, Alberto Verrotti, Martino Ruggieri, Paola Iannetti

**Affiliations:** 1 Child Neurology, Paediatric Department, I Faculty of Medicine, “Sapienza University”, c/o Policlinico Umberto I, Rome, Italy; 2 Child Neurology, Headache Paediatric Center, Paediatric Sleep Centre, Chair of Paediatrics, II Faculty of Medicine, “Sapienza University”, c/o Sant’Andrea Hospital, Rome, Italy; 3 Child Neurology, Pediatric Department, University of Chieti, Italy; 4 Child Neurology, Pediatric Department, University of Catania, Italy

**Keywords:** Demyelinating disease, pediatric multiple sclerosis, ADEM, immune-mediate polyradiculoneuropathies, disease-modifying therapies, immunomodulatory therapy.

## Abstract

Inflammatory demyelinating diseases comprise a spectrum of disorders affecting the myelin of the central and peripheral nervous system. These diseases can usually be differentiated on the basis of clinical, radiological, laboratory and pathological findings.

Recent studies have contributed to current awareness that inflammatory demyelinating diseases are not restricted to the adult age group, but are more common in pediatric age than previously believed. Some of pediatric inflammatory demyelinating diseases carry an unfavorable long-term prognosis but appropriate treatments can improve the outcome. The possibility of physical and cognitive disability resulting from these diseases, highlights the urgent need for therapeutic strategies for neurorehabilitation, neuroregeneration, and neurorepair.

This review discusses characteristics of primary demyelinating diseases more frequently observed in childhood, focusing on epidemiology, clinical aspects and treatments.

## INTRODUCTION

Inflammatory demyelinating diseases comprise a spectrum of disorders affecting the myelin of central (CNS) and peripheral nervous system (PNS). These disorders can usually be differentiated on the basis of clinical, imaging, laboratory and pathological findings [1]. 

Demyelination is often secondary to an infectious, ischemic, metabolic, or hereditary disorder. The cause of primary demyelinating disorders is unknown, but autoimmune mechanisms are suspected [2]. The concept of primary demyelination implies the destruction of myelin sheets, oligodendrocytes, and Schwann cells with relative preservation of other nervous system components. However, axonal injury is a common finding in demyelinating lesions, which well correlates with permanent functional deficits [3].

Some myelin disorders tend to affect primarily the myelin of peripheral nerves, while others affect the CNS. In most individuals, demyelinating diseases are restricted to one or the other compartment but patients with concomitant CNS and PNS inflammatory demyelinating processes have been reported [4, 5].

Idiopathic inflammatory-demyelinating diseases (IIDDs) constitute a heterogeneous group of CNS disorders thought to be of autoimmune origin and including acute disseminated encephalomyelitis, multiple sclerosis, Devic’s disease, transverse myelitis, and clinically isolated syndromes such as optic neuritis. This spectrum includes monophasic, multiphasic, and progressive disorders ranging from highly localized forms to multifocal or diffuse variants (Table **1**) [6, 7]. 

Acquired inflammatory demyelination of the peripheral nervous system also may have autoimmune origin and can present acutely as the heterogeneous entity known as Guillain-Barre´ syndrome (GBS) or in a more protracted, sometimes relapsing course, known as chronic inflammatory demyelinating polyradiculoneuropathy (CIDP) (Table **2**) [5]. 

Recent studies have contributed to current awareness that these disorders are not restricted to the adult age group, but are more common in the pediatric age then hitherto believed. Some of pediatric inflammatory demyelinating diseases carry an unfavorable long-term prognosis but timely and tailored treatments can improve the outcome. The possibility of physical and cognitive disability resulting from these diseases, highlights the urgent need for therapeutic strategies for neurorehabilitation, neuroregeneration, and neurorepair [3]. The therapeutic strategy is actually in progress. Actually it is defined that acute demyelination in children is managed in the same way as adult patients, while DMTs have not been approved by FDA for treatment of pediatric demyelinating diseases [8, 9]. However, encouraging data regarding safety and efficacy of first-line therapies come out from the few study on pediatric population.

This review discusses characteristics of primary demyelinating disease more frequently observed in childhood, focusing on epidemiology, clinical aspects and treatments.

## MULTIPLE SCLEROSIS

Multiple Sclerosis (MS), the prototype of the autoimmune inflammatory diseases of the CNS, is characterized by both axonal damage and diffuse areas of inflammatory non-vasculitic demyelination involving the encephalic and spinal white matter [10]. 

To date, the pathogenesis of MS is not completely explained and, as the other autoimmune diseases, it is considered to be a complex interplay of immune system reactivity and environmental triggers (e.g., viruses) associated with a background of genetic susceptibility resulting in an immune-mediated demyelination, axonal loss, and neurodegeneration. Putative environmental triggers, such as vitamin D homeostasis, early dietary exposures, seasonality of birth, and the timing and sequence of viral exposures during childhood are being explored [8]. 

MS primarily affects young adults, with a peak of incidence between the ages of 20 and 40, and is the main cause of non-traumatic neurological disability in the industrialized world [11]. However, it is estimated that, in approximately 1% of cases, the onset of MS (defined by the presence of the first neurological symptom) is before 10 years (very early onset multiple sclerosis, VEOMS) and up to 3-5% between 11-16 years (early onset multiple sclerosis, EOMS) [12, 13]. The term “Pediatric MS” refers to the first clinical demyelination episode before the age of 18.

The female preponderance observed in adult-MS is also notable in pediatric-MS, but the female:male ratio in patients with pediatric-MS is approximately equal. A family history of MS is present in 6-8% of children with MS [14].

The diagnosis of MS in pediatric age is not easy to achieve because of difficulties for clinicians in recognizing the patients that do not fit the typical age range of 20-40 years [15]. In addition, the trouble of the differential diagnosis is linked to other inconveniences such as the many types of primary or secondary CNS disorders resembling demyelinating diseases, or the atypical presentation of clinical (fever, involvement of peripheral nervous system, encephalopathy, absence of attacks symptoms, progressive course), laboratory [absence of cerebrospinal fluid (CSF) oligoclonal IgG, CSF pleocytosis, elevation of leukocyte count or erythrocyte sedimentation rate], neuroimaging findings [16, 17]. Thus, MS is probably underdiagnosed in the pediatric population [11]. 

Among the numerous groups of diseases that may lead a misdiagnosis of MS we distinguish infections (neuroborreliosis, neuro-AIDS, neurosyphilis, subacute sclerosing panencephalitis, parasites, progressive multifocal leukoencephalopathy, Human T-cell lymphotropic virus), neoplasms (astrocyroma, lymphoma), inflammatory diseases (systemic lupus erythematosus, Bechet disease, neurosarcoidosis, Sjogren disease), leukoencephalopathies (adrenoleukodystophy, metachromatic leukodystrophy, Krabbe disease, Pelizaeus-Merzbacher disease, Refsum disease, MELAS, MERFF, Wilson disease, Alexander disease, Fabry disease, Leigh disease, Kearns-Sayre syndrome), vitamin deficiencies (B12 or E or folate deficit), gastrointestinal disease (Whipple disease, celiac disease), vascular disorders (moya-moya disease, CADASIL, vasculitis), and other disorders [17].

In order to improve our understandings on pediatric demyelinating diseases and to better characterized the clinical features [18], the differential diagnosis [17] and optimal therapy [9] for these disorders, the International Pediatric MS Study Group developed consensus definitions regarding the major CNS inflammatory demyelinating disorders of children and adolescents [19]. 

As for adult patients, the diagnosis of pediatric MS requires dissemination in space and time, involving the necessity of, at least, a second episode before establishing it. The space involvement could be evaluated by means of both a history and neurological findings consistent with a multifocal disease, and neuroimaging techniques [11]. The MRI is used in the first three months after the initial clinical attack (also called Clinically Isolated Syndrome), and it is considered positive if three of the following four features are satisfied [20]: 1) nine or more white matter lesions or one gadolinium enhancing lesion, 2) three or more periventricular lesions, 3) one juxtacortical lesion, 4) an infratentorial lesion [19]. Two MRI lesions, with one in the brain, are sufficient for meeting dissemination in space criteria if associated with abnormal (CSF) (e.g. oligoclonal band or elevated IgG index) [19]. However, at present, there are four proposed MRI diagnostic criteria for pediatric MS needing for validity [20-23].

Finally, the ADEM diagnostic criteria should not be achieved (see ADEM section for ADEM diagnostic criteria and for comparison with MS) [19]. If first episode is ADEM, two or more non-ADEM events are required for diagnosis of MS. New MRI lesions 3 months or longer after the initial clinical event can be used to satisfy criteria for dissemination in time [19]. 

**Fig. (1):**
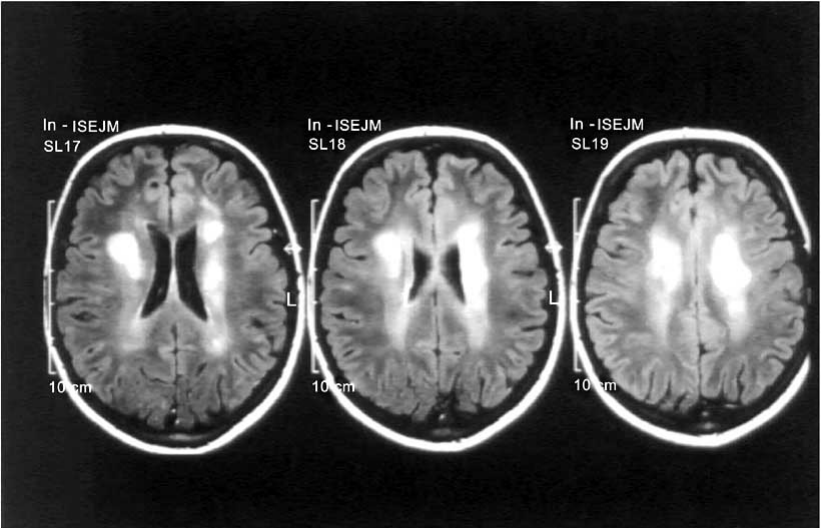
Axial view T2 weighted. A 12-year-old girl coming to our attention for acute right hemiparesis and deficiency of the seventh cranial nerve. Multiple lesions in the periventricular white matter prevailing on the left side. Diagnosis: tumefactive MS.

The main clinical features of MS encompass visual, sensory, motor and cognitive functions, although every neurological functions may be involved in connection with site and/or size of the active demyelinating lesions (e.g. lesions bigger than or equal to 2 cm have been renamed “tumefactive demyelinating lesions” (Fig. **1**) and may be clinically devastating [24], with encephalopathy and seizures). Pediatric patients typically have a polysymptomatic onset, although monosymptomatic presentations are not uncommon [25]. 

The natural history of MS is variable, and 4 different sub-types have been recognized on the basis of the clinical course in pediatric population: Relapsing-Remitting (RR) form, occurring for 90% of cases, is characterized by periods of neurological dysfunctions followed by clinical improvement (partial or complete) and spaced out by clinical stability; the remaining patients could experience Primary-Progressive form (PP; 2.3-7% of cases), Secondary-Progressive (SP) form (as evolution of a RR form, consequence of a progressive neurological function deterioration, even in the absence of acute events [26]), Progressive-Relapsing (PR) form [18]. In the initial stages, pediatric MS is considered to have a more favorable prognosis compared to adult MS; however, over the long term, pediatric MS patients can become disabled at younger age owing to the periodic demyelinating episodes, indicating that MS in pediatric population does not have a benign outcome [18]. Consequently, a well-timed and correct diagnosis of early MS is pivotal for starting the appropriate treatment and management [9]. It should be kept on mind that adolescents with MS have an increased risk of depression, mood-swings and behavioral problems, that could be raised by the disease course, the onset of disability, the use of disease-modifying therapies [27]; thus, the management should be multidisciplinary, involving neurologist and psychologist or psychiatrist [25]. 

The management of acute relapses in children generally starts with intravenous corticosteroids, at high doses of 20-30 mg/kg/day (maximum 1 g per day) for 3-5 days, often resulting in dramatic clinical improvement [25]. Oral prednisone, starting at a dose of 1 mg/kg/day and tapered over 14-21 days, is administered in cases of incomplete resolution of symptoms after intravenous therapy [25]. 

The most frequent side effects of high-doses corticosteroids are facial flushing, sleep difficulties, irritability, increased appetite, and, especially in children needing for prolonged administration, growth retardation. Side effects such as high blood pressure and hyperglycemia are rare in pediatric age, but corticosteroids therapy requires careful monitoring of blood pressure, urine glucose, serum potassium, and co-administration of gastric protection. The risk of side effects is directly proportional to prolonged use and total cumulative dose; reviewed doses are generally well tolerated, even when a short oral taper dose is given [9].

Intravenous immunoglobulin (IVIG) (1 g/kg monthly or every 3 months, with or without a 5 day induction of 0.4 g/kg daily, for a total time ranging from 6 to 12 months) or plasma-exchange could be used acutely in children who fail to respond to corticosteroids [9, 25] or fail, decline, or are not able to take standard immunomodulatory therapies [28]. The meta-analyses and the several randomized controlled trials on adult MS population suggest that IVIG are better than placebo in patients with RR-MS, but no trials comparing IVIG against standard treatment and DMT in pediatric MS patients are available [28]. Furthermore, IVIG in association with high-doses steroids doesn’t seem better than steroids alone to treat relapses [9]. IVIG efficacy in pediatric population remains unclear [9, 25, 29], and thus, IVIG is not recommended for routine treatment of primary or secondary progressive MS [28]. However, IVIG could be considered as option for treatment of patients with severe, refractory optic neuritis which have had no recovery of vision after 3 months of standard steroid therapy [28]. The management of relapses with plasma-exchange is proposed in adult-MS with severe relapses, after the failure of therapy with high-doses steroids; thus, plasma-exchange has been suggested in pediatric-MS if the gap between acute episodes is too restricted, that hinders the high-doses glucosteroids administration and makes easier the onset of adverse effects [9]. To date, no studies regarding plasma-exchange efficacy and utility in pediatric MS are available.

In addition to the mentioned options, several disease-modifying therapies (DMT) are available for pediatric multiple sclerosis: immunomodulatory and immunosuppressive drugs belong to these therapies. DMT can reduce the duration of symptoms or modify the progression of the disease [14, 30, 31]. Although open-label studies of DMT such as interferon beta-1a [32, 33], interferon beta-1b [31, 34] and glatiramer acetate [31, 35] have demonstrated safety and efficacy for children, these drugs have not been formally approved for pediatric use. There is Class I level of evidence for these medications by means of a reduction in frequency of clinical relapses (approximately 30%) and reduction in disease activity seen on brain MRI. Adverse effects such as flu-like symptoms, injection site reaction, elevated liver enzymes, or a perceived lack of efficacy by the adolescents may result in non-adherence to treatment [27]. 

The starting doses for interferon therapy in pediatric MS are object of discussion; however, it is well-know that is pivotal to monitoring liver function and white blood cell counts monthly; younger children may have a transient elevation in liver transaminases that necessitates a slower escalation to full dose [8].

Glatiramet acetate (GA) and Interferon-beta 1a (IFNβ-1a) and 1b (IFNβ-1b) belong to the group of immunomodulatory therapy approved for adults Relapsing-Remitting MS, on the basis of their ability to reduce MRI lesions and relapses rate [9].

IFNβ-1b is a lyophilized protein produced by DNA recombinant technology by E. Coli with a wide series of effects on the immune systems such as inhibition of T-lymphocyte proliferation and decrease of IFN-γ production, inhibition of major histocompatibility complex class II (MHC II) expression, induction of anti-inflammatory and inhibition of pro-inflammatory cytokines [36]. It has been used in children at the same adult dose (8 Million International Units, MIU) [34]. The most frequent adverse effects encompass flu-like manifestations, altered liver function test and injection side reaction, without dangerous unforeseeable adverse events [34]. However, to date, although IFNβ-1b treatment has very favorable efficacy on clinical and MRI measures of disease activity and progression from the very early stages of disease, numerous studies on long-term efficacy and safety in pediatric ages are not available [9, 31]. 

Interferon beta-1a Intramuscular has been administrated in pediatric MS in few studies [32, 37-40] at full doses of 30 µg / week or lower (15 µg / week) in children <10 years or with a body weight less than 30 kg [25], showing in some cases [39] the necessity of treatment interruption for adverse effects besides flu-like symptoms, headaches, fever, and injection site soreness. However IM IFNβ-1a seems to be effective and well tolerated in pediatric patients with MS [32].

IFNβ-1a could be used subcutaneously at the 1/2 dose of 22 µg times per week (tiw), but a relapse necessitates an increase at the full dose of 44 µg three times weekly [9, 33, 41]. Still for IFNB-1a mild/moderate or severe adverse effects such as systemic reaction and depression are described [33]. 

Glatiramer acetate (GA) is made of a random polypeptide (L-glutamic acid, L-lysine, L-alanine, and L-tyrosine), which induces an antigenic cross-reactivity with myelin protein reactive T cells. GA induces the production of anti-GA-regulating Th2 T cells that regulate CNS inflammation [11]. GA is administered daily as 20 mg subcutaneous injections [9], and seems to be well tolerated [37, 42]. Monitoring of liver function and hematological parameters is not required [8].

The immunosuppressive therapy is generally reserved to children with severe course, frequent relapses, progression of disability and not responding to first-line treatment, although pediatric literature data regarding efficacy and safety are not sufficient [3, 8]. Azathioprine, mitoxantrone, cyclophosphamide or methotrexate have been used as escalation treatment of pediatric MS, either as monotherapy or as add-on to immunomodulation [3]. As well for immunomodulatory medicaments, also the immunosuppressive drugs group has not been approved by US Food and Drugs Administration (FDA) for MS, with the exception of mitoxantrone for adult patients [11].

Azathioprine has been used at oral dose of 1-2 mg/kg/day [11] in order to avoid relapses, for patients that do not respond or tolerate immunomodulatory drugs [9, 11]. Disadvantageous effects encompass cytopenia, skin rashes, gastrointestinal intolerance, abnormal liver toxicity, requiring periodic blood count and liver functions [9, 11]. No certain data confirm the risk of secondary cancer after azathioprine use [9, 11].

Mitoxantrone is administrated quarterly in adults at doses of 12 mg/m^2^ IV (maximum cumulative dose of 120 mg/m^2^) for a full period of 2 years [11], showing good results in terms of decreases in progression of disability, relapses rate, and onset of new T2 weighted MRI lesions, compared with placebo. Cardiotoxicity, leukemia, amenorrhea and infection are expected effects. For these reasons, the indication of mitoxantrone is limited to cases of worsening adult MS resistant to other treatments [43]. There is no published experience of mitoxantrone in the pediatric MS population [9, 11]. 

Cyclophosphamide is an alkylating agent used mainly as a second-line treatment in multiple sclerosis, and several studies have suggested this drug as most beneficial in younger adult patients and in patients with early secondary progressive MS [11]. Very recently it has been described a retrospective study on its use in pediatric series [44]. Cyclophosphamide, administered at 600 to 1, 000 mg/m^2^ per dose, with the minimum dose required to achieve lymphopenia (nadir total white blood cell count less than 3, 000/mm^3^ or between 1, 500 and 2, 000/mm^3^, depending on the institution) used to guide maintenance therapy, reduces relapses rate and stabilizes disability scores in the majority of patients [44]. The entire period of therapy could range from few months to 3 years, although its use is limited by the onset of adverse effects or risk of cystitis and neoplasm [9]. Its effects on MRI lesions remains undefined. Infections (especially with white blood cells < 1500/mm^3^), nausea, transient alopecia, gonad failure, hemorrhagic cystitis, bladder and blood cancers (particularly if cumulative cyclophosphamide dosage is 100 g or more [44]), could derive from treatment with cyclophosphamide [9, 11, 44]. 

Methotrexate is rarely used in pediatric MS, and only occasionally in progressive forms of MS in adult patients. The dosage is 7, 5-20 mg per os once per week, in association with folic acid (1 mg/day) to prevent macrocytic anemia. Other major side effects are liver and lung toxicity [9, 11].

Natalizumab, a humanized monoclonal antibody directed against alfa-4 integrins [45] and approved for acute adults RR-MS type (300 mg infused monthly) [9], may be another medical option in pediatric MS. Recently it has been administrated at a dosage of 3 to 5 mg/kg of body weight every 4 weeks in three children with a previous poor response to immunomodulatory medicine or with excessive drugs reaction [46]. All these patients had no further relapses, all reported significant improvement in quality of life, and no new T2-weighted lesions or gadolinium-enhancing lesions were detected at MRI follow-up [46]. Finally no unpredictable events were seen when dosage was adjusted to body weight [46]. Therefore, it is a promising second-line therapy for pediatric patients with RRMS unresponsive to immunomodulatory therapy, although not licensed for patients under the age of 18 years.

In conclusion, to date, the therapeutic strategy of pediatric MS is complicated and variable in relation with the clinical course. The relapse therapy is based primarily on IV corticosteroids, and, in second instance IVIG or plasma exchange. The DMT is approved in case of active RR disease, clinically or by MRI defined (more than one clinical relapse or new T2 weighted lesions or gadolinium enhancing lesions in a period of 1 to 2 years). First line DMT include GA, IFNB 1a and 1b, the sole approved by FDA. The drugs should be chosen in co-operation with the patient and his family after a complete illustration of efficacy, tolerability and risks. It has to be kept on mind that clinical, immunologic, pharmacokinetic, and pharmacodynamic responses to treatment are different in children versus adults, for example in liver and kidney function, fat distribution, gut absorption, and availability of adequate subcutaneous tissue for injection. Thus, the dosage should be adjusted in relation with body weight, and, eventually, on the basis of follow-up data. The follow-up should include laboratory, MRI scans acquisitions, clinical examinations, at a time depending on the used drugs. In general, during the first month, liver function and total blood count should be carefully checked. The International Committee suggest that brain MRI scans should be acquire 6 to 12 months after starting DMT, and compared to the older scans. Neurological examinations should be performed at baseline and then at 1, 3, 6 months, and continued every 6 months [19]. After the first year of therapy in stable patients without new attacks, clinical and neuroradiological follow-up can be performed yearly. In case of first-line treatment failure, addition of IV corticosteroids pulse and the second-line drugs should be taken into account. 

A better understanding of the immunologic and pathophysiological mechanisms underlying multiple sclerosis have recently led to the design of numerous novel medical approaches, based on selective and target-specific therapies instead of indiscriminate immunosuppresion or global immunomodulation, and trying to protect neurons against axonal damage and loss [47-49].

However, there are unmet needs in MS treatment, such as the absence of oral formulations and the adverse side effects that mean the partial efficacy, safety and tolerability of actual DMT [47].

## ACUTE DISSEMINATE ENCEPHALOMYELITIS

Acute disseminate encephalomyelitis (ADEM) is a multifocal inflammatory disease of Central Nervous System with a benign evolution, usually appearing after an infection or a vaccination. ADEM is often characterized by a monophasic course with multiple neurological symptoms, including encephalopathy-like syndrome [50-52], which could be absent in 0-18% of ADEM patients [8]. Initial symptoms depend on the size and the areas involved by the lesions; consequently the wide clinical spectrum comprises unilateral or bilateral pyramidal signs (60-95%), hemiplegia (76%), ataxia (18-65%), cranial nerve palsies (22-45%), spinal cord involvement (24%), seizures (13-35%), optic neuritis (7-22%), speech alterations (5-21%), hemiparesthesia (2-3%). The most of symptoms occur in both adults and children, although some of them seem to be age-related: long-lasting fever, headaches and seizures are more frequently observed in pediatric patients, while sensory alterations and PNS involvement (acute polyradiculoneuropathy) in adults [53]. ADEM pathogenesis is not completely defined; either the post-infective or post-vaccinous onset, and the presence of autoantibodies against the tetrameric myelin oligodendrocyte glycoprotein (MOG), support the hypothesis of inflammatory demyelination triggered by autoimmune mechanisms [3]. Although the disease is defined as acute, not necessarily all the neurological manifestations have to show themselves at the same time; in fact, new symptoms or signs, occurring within the first 3 months from the initial episode, are considered to be part of the same attack [8]. More commonly children less then 10 years of age are affected [14], with a mean age range of 5-8 years [53]. The gender ratio appears to be quite balanced, with slight male preponderance [3]. Rarely relapses can occur, configuring a picture of multiphasic or recurrent ADEM. In multiphasic ADEM new clinical attack involves CNS areas previously uninjured at least 3 months after the first event and at least 1 month after the end of steroids therapy [53]. Differently, the term recurrent ADEM is used if the relapse, always arising at least 3 or more months after the first event and at least 1 month after the end of steroids therapy, has the same neurological and neuroradiological features, suggesting an unmodified involvement of CNS areas [53]. However, both in case of multiphasic and recurrent type, the ADEM criteria should be met [8]. Actually, accurate diagnostic criteria for ADEM are lacking, owing to the absence of specific markers [53]. Thus, the main difficulty is the differential diagnosis with pediatric Multiple Sclerosis, especially in cases of multiphasic or recurrent ADEM, whose correct identification may require a long-term follow-up [53]. ADEM diagnostic criteria proposed by an International committee include encephalopathy (defined as mental status changes and/or behavioral alterations) and polysymptomatic presentation as cardinal point for diagnosis [19]; MRI and CSF findings, documentation of a prior infection and clinical evolution could be useful if associated with the previous cardinal criteria [19].

**Fig. (2):**
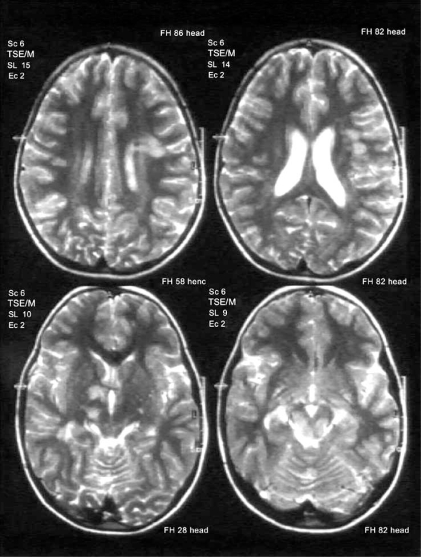
Axial view T2 weighted: A 7- year-old boy who came to our attention because of headache and lethargy following one day of fever. Multiple lesions in the left temporo-parietal white matter junctions, in the right thalamus and in the pons. Diagnosis: ADEM.

Some clinical, neuroradiological and CSF findings could be useful in formulating differential diagnosis with MS. For example, the ADEM clinical onset is frequently polysymptomatic, with consciousness and behavioral changes (encephalopathy, typically not common in MS), and rarely with optic neuritis and sensibility disturbances, typical of MS onset [53]. The Magnetic Resonance Imaging, particularly T2-weighted and fluid-attenuated inversion recovery (FLAIR) sequences, shows lesions of the subcortical, juxtacortical and central white matter of hemispheres, cerebellum, periventricular zone, brainstem, spinal cord (Fig. **2**) [53]. The gray matter of thalami and basal ganglia is often affected [53]. The lesions reported above are typically described as highly variable in size (more frequently large), multiple, bilateral, asymmetric [50, 53]. The gadolinium enhanced T1-weighted sequences could show lesions in a variable percentage of patients, depending on the inflammatory state [53]. These neuroradiological features of size and distribution could be very significant in distinguishing an ADEM from MS attack [50]. However, the MRI pattern described for ADEM are numerous [54]. Finally, CSF analysis could demonstrate an increased number of protein and lymphocyte (more then 30 cells/all), although normal values or IgG oligoclonal bands may result [50]. There is not a well-defined standard treatment for ADEM [53]. Treatment of demyelinating attacks is based on intravenous corticosteroids administration (Methylprednisolone at 10-30 mg/kg/day for 3-5 days, with a maximum dose of 1 g daily; dexamethasone at 1 mg/kg) during acute phase, aiming to reduce the inflammatory process. Subsequently, a maintenance steroids oral taper, during 4-6 weeks, is pivotal to decreasing the risk of recurrence [3]. Better outcome seems to be associated with methylprednisolone compared to dexamethasone treatment, although this comparison has been investigated only in one study [54], and it has not been confirmed. The adverse effects are the same described for high-doses steroids therapy in MS. Intravenous immunoglobulins, at doses of 1-2 g/kg for a period of 2-5 days, are considered effective and recommended in children with persistence of symptoms or steroids inefficiency [3]. Not many data are reported in literature about plasmapheresis use in ADEM patients, and it is considered a rescue treatment after the failure of steroids and IGIV, although it seems to be more useful if administrated in earlier stage of disease [55]. Up to date, DMT are not reported to be used in children with ADEM [53]. 

## SCHILDER’S DISEASE

Schilder's disease, or myelinoclastic diffuse sclerosis, is a rare acute or subacute demyelinating disorder of central nervous system affecting children and young patients. 

In the past this idiopathic inflammatory demyelinating disease was considered a subtype of Multiple Sclerosis; recent data have demonstrated that this entity presents different clinical, radiological, laboratory and pathological findings from other demyelinating diseases (Table **3**) [6].

In 1912, Schilder first described the disease as “encephalitis periaxialis diffusa” and until 1985 the term Schilder’s disease was used to describe different demyelinating disorders of different etiologies, including adrenoleukodystrophy and subacute sclerosing panencephalitis [56].

In 1986, Poser established the diagnostic criteria for non invasive diagnosis of Schilder’s disease: clinical symptoms and signs often atypical for the early course of MS, CSF normal or atypical for MS, large bilateral areas of demyelination of cerebral white matter, no fever, no viral or mycoplasma infection, nor vaccination preceding the neurological symptoms and normal serum concentration of very long-chain fatty acids [57].

Pathologically, the disease is characterized by diffuse inflammatory demyelination similar to MS, with areas of demyelination are grossly more extensive, leading to the term “myeloclastic diffuse sclerosis”. The relationship between Schilder’s disease and MS is unclear, although patients with Schilder’s disease may develop a relapsing-remitting courses, typical of MS, and considered by Poser a disease “in transition” between Schilder’s disease and MS (“transitional sclerosis”). Differences include the apparently more severe and often progressive course, and lack of pleiocytosis and oligoclonal band in CSF in patients with Schilder’s disease. However the distinction between MS and Schilder’s disease is probably not so clearly defined [6].

The underlying cause of Schilder's disease is unknown. Symptoms are caused by widespread patches of demyelination throughout the brain and spinal cord, resulting in slowed, faulty nervous transmission.

Most patients with Schilder's disease are diagnosed between the ages of 5 and 14 years. Patients present with gradually more severe symptoms and the disease course can include psychiatric predominance, dementia, emiplegia, aphasia, ataxia, intracranial hypertension, cortical blindness, deafness and other symptoms such as seizures, tremors, poor attention, headache, muscle weakness, vomiting, impaired vision, personality changes, and difficulty keeping balance [58]. Some children have a relentless progressive course, culminating in death. Other children may have remissions and exacerbations, with each subsequent exacerbation more severe and each remission less complete than previous episodes, until death supervenes [59, 60]. Schilder's disease is uniformly fatal [60, 61]. The prognosis of Schilder’s disease is very variable and can take three different courses: monophasic not remitting, remitting, and finally, progressive with increase in deficits.

Imaging studies show large ring-enhancing lesions involving both hemispheres, sometimes symmetrically and located particularly in the parieto-occipital regions, tipically confined to the centrum semiovale [6]. The differential diagnosis with brain tumors or abscesses of these large demyelinating brain lesions is often difficult, and invasive procedures, such as biopsy, are still the gold standard for diagnosis [57]. 

Since Schilder's disease is similar to multiple sclerosis, treatments for this disease are similar and include high-doses steroids, beta interferon, immunosuppressive and symptomatic therapy. The treatment team for children with Schilder's disease usually consists of neurologists, specialists in multiple sclerosis, and rheumatologists. Support from physical therapists, occupational therapists, and speech and language therapists can help children in preserving functionality as much as possible.

Treatments aim to slow the inexorable course of the disease. Corticosteroids, such as Methylprednisolone IV, are used in high doses and often long courses to control the disease. This treatment results in some improvement in the majority of patients. Immunoglobulins have been used with questionable effects in one case and without effect in two patients [60, 62].

Other drugs used were Cyclophosphamide followed by Azathioprine in a 12-year old boy, reported to be stable on this regime, whereas Azathioprine did not prevent the progressive decline in a 12 year old girl [62].

In another patient the use of Ciclophosphamide in combination with ACTH was followed by complete recovery. Recent studies about therapy in pediatric patients with Schilder’s disease are lacking and the therapy is based on both anecdotal reports of the treatment and recommendations for MS [60, 61].

## NEUROMYELITIS OPTICA OR DEVIC’S SYNDROME

Neuromyelitis optica (NMO) or Devic’s Syndrome is another idiopathic inflammatory demyelinating diseases of the central nervous system in differential diagnosis with MS. 

It has been long debated whether neuromyelitis optica is a variant of MS or a separate disease, because their clinical features are similar: optic neuritis, myelitis and inflammatory demyelination. Recently Devic’s syndrome has been recognized as a discrete, relapsing, demyelinating disease with different clinical, radiological and laboratory findings from MS [63].

The first case was described in 1894 by Device and Gault, as a disease characterized by optic neuritis and acute transverse myelitis. Regarding etiology, the cause of the disease is unknown, but various previous or coexisting infections (tuberculosis, viral infections), vaccinations and systemic autoimmune diseases (LES, Sjogren syndrome, rheumatoid arthritis, Hashimoto thyroiditis) have been associated with NMO [63, 64].

NMO is the first inflammatory autoimmune demyelinating disease of the CNS for which a specific antigenic target, the astrocytic water channel aquaporin-4 (AQP4), has been identified. This protein is located on conglomerated astrocyte foot processes, which abut cerebral microvessels of the blood-brain barrier. An IgG specific autoantibody for this water channel, called NMO-IgG, has been recently discovered and used as a clinically validated serum biomarker for the differential diagnosis between NMO and MS. It can be detected by an indirect immunofluorescence assay [64, 65]. It has been suggested that, in susceptible individuals, an antigenic trigger stimulates production of circulating immunoglobulin, NMO-IgG, which are able to reach their target antigen (aquaporin-4) through a breach in the blood-brain barrier. The binding of antigen-antibody and the activation of complement lead to an inflammatory response. The disruption of the cellular water transport mechanisms and the inflammatory necrosis may explain the radiologic and pathologic findings in NMO [64]. NMO is more frequent in women than in men (>80% of cases) [66] and affects young adults, but throughout the twentieth century children affected by NMO were recorded. The average age of onset is 29 years for monophasic patients and 39 years for relapsing patients. The prevalence and the incidence of this syndrome is unknown [66, 67]. The attacks of neuritis are more commonly unilateral than bilateral, but the presence of bilateral simultaneous optic neuritis is a hallmark of NMO and rare in MS. The attacks of optic neuritis and myelitis usually occur sequentially rather than simultaneously. Complete loss of vision, ocular pain with eye movements, incomplete recovery and myelitis with severe symmetric paraparesis or quadriparesis, loss of sensation below the site of inflammation and bladder and bowel retention or incontinence are typical features of NMO. Other symptoms of spinal cord demyelination consist of paroxysmal tonic spasms and Lermitte’s symptom (spinal or limb dysaesthesias caused by neck flexion); cervical myelitis can damage the brainstem, causing nausea, vertigo, vomiting, hiccups, diplopia, ataxia or acute neurogenic respiratory failure [63, 67, 68]. Today diagnosis of NMO requires absolute criteria ( optic neuritis and acute myelitis) and at least two out of three supportive criteria ( negative brain MRI at disease onset, spinal cord MRI with contiguous T2-weighted signal abnormality, extending over 3 or more vertebral segments and NMO-IgG seropositive status) [65]. The natural history of NMO is variable; there are two courses of the disease, monophasic or relapsing. Most patients (75%) with NMO develop a relapsing course with recurrent optic neuritis and myelitis. Optic neuritis can be separeted from transverse myelitis by months or years, and episodes tend to relapse with significant increasing in disability. The monophasic course occurs in about 25 % of patients, with concomitant involvement of either unilateral/bilateral optic neuritis and a single episode of transverse myelitis. In these patients a longitudinal follow-up over years does not reveal new attacks [66, 68]. In regard to treatment, it’s necessary to distinguish a therapy for the acute attack and a therapy for the relapses. In the first case hospitalization is commonly required, particularly when acute ventilatory failure occurs. Other manifestations related to myelitis are deep vein thrombosis, pulmonary embolism, autonomic dysfunction, deceits ulcers and infections. So the affected patients often require long term recovery in rehabilitative units [66]. Therapy for the acute attacks consists of Methylprednisolone IV for 5 days ( 30 mg/kg/day under 30 kg body weight to 1 g/day over 30 kg body weight) [9, 66]. In acute attacks refractoring to steroids, plasma-exchanges (55 ml/kg every other day) and IV Immunoglobulins (0, 4 g/kg once a day for 5 days) have also been used and may be somewhat helpful to children. Thus plasmapheresis is recommended for patients with severe attacks that worsen during corticosteroids therapy or do not show improvement [9, 69]. In regard to relapsing disease, in a study of seven patients, the therapy included a maintenance combination treatment with Azathioprine (2, 5-3 mg/kg/day) and Prednisone (1 mg/kg/day). After two months the prednisone dose was gradually reduced until a maintenance dose of 10 mg/day. During the follow-up period no attacks were recorded and the disability score was improved [66]. Some data suggest that Interferon and Glatiramer Acetate are not effective in relapsing NMO disease. Other studies demonstrate the effectiveness of Mycophenolate mofetil, Prednisone, Rituximab, Mytoxantrone and Cyclophosphamide in these patients [68, 70, 71].

## GUILLAIN BARRÈ SYNDROME

Guillain Barrè syndrome (GBS) is an inflammatory autoimmune disorder affecting the peripheral nervous system in which segmental demyelination and conduction block are the pathological and electrophysiological correlates of muscle weakness [72]. Different subtypes producing the clinical picture of GBS have been described including acute inflammatory demyelinating polyradiculoneuropathy (AIDP), acute motor axonal neuropathy (AMAN), acute motor and sensory neuropathy (AMSAN), acute sensory neuronopathy, acute pandysautonomia and the Miller- Fisher syndrome [73]. 

GBS incidence is about 0.5-1.5 per 100, 000 people younger than 18 years of age. Males are about 1.5 times more likely to be affected than female. AIDP is the most common GBS subtype in North America, Europe and Australia affecting all ages (about 85-90%). In northern China, Japan, Asia, Latin America and the developing world axonal forms occur more frequently. Atypical cases such as polyneuritis cranialis and Miller- Fisher syndrome are much less common and reported in 20 of 179 patients (11.2%) [5]. 

Antecedent events such as infections and vaccinations occur 1-6 weeks before onset in about 50-82% of children [5]. Some of the pathogenic triggers of GBS include viruses such as HIV, Epstein-Barr virus, Cytomegalovirus, Hepatitis and Varicella. Campylobacter Jejuni infection often with diarrhea is the most common identifiable infectious precedent of GBS, particularly with the Miller-Fisher variant. Campylobacter jejuni was also found to be responsible of summer epidemics of the AMAN form of GBS in China [73]. Mycoplasma pneumoniae also remains an important antecedent infection responsible for GBS [74]. GBS has also been reported following surgery and head trauma [75]. 

The disorder is characterized by symmetrical weakness which usually begins in the lower extremities and progressively involves the trunk, the upper limbs, and finally the bulbar muscles. Classically, both proximal and distal muscles of limbs are involved in AIDP simultaneously [73]. The weakness has a gradual onset and reaches its worst within four weeks evolving in walking difficulties and later in flaccid tetraplegia [76]. Reflexes are usually lost early in the illness, although GBS can be associated with retained reflexes or even brisk reflexes [76]. Sensory loss, if present, usually takes the form of loss of proprioception while loss of pain and temperature sensation is usually mild. In fact, pain is a common symptom in GBS, presenting as deep aching pain which patients compare to the pain from over exercising. Pain usually affects the weakened muscles but it is often localized in the neck and back. In some cases patients complain paresthesias [5]. 

The cranial nerves are often involved, and facial weakness and bulbar palsy are the most common problems, followed by eyes movements disorders [76]. Oropharyngeal dysphagia and facial weakness interfere with swallowing, drooling, and/or maintaining an open airway [78]. 

Respiratory muscle weakness may be severe enough to warrant artificial ventilation in about 25% of patients and portends a poor prognosis [73]. Dysautonomia occurs in 15% of patients manifesting as cardiac arrhythmia, hypertension or hypotension, ileus and urinary retention [73]. 

Although the axonal forms of GBS appear similar to AIDP, there are important clinical differences. Specifically, the axonal forms of GBS exhibit a more rapid and severe course, with frequent respiratory involvement and ventilator dependence, along with cranial nerves involvement and infrequent and mild involvement of the autonomic nervous system. The AMAN form is a pure motor syndrome, with rapid onset of muscle weakness and absent reflexes, while AMSAN is clinically characterized by the presence of both motor and sensory deficits [73]. 

Miller-Fisher syndrome is a focal form of GSB consisting of acute external ophthalmoplegia, ataxia and areflexia [77]. The course is similar to that of typical GBS and recurrences are quite rare [74]. 

Although GBS reaches a nadir at 2 weeks to 4 weeks, with most patients recovering from this debilitating illness, 10% to 20% of patients are left with disabling motor deficits and 4% to 15% of patients die by 1 year after onset [5, 73]. 

Prolongation of worsening weakness beyond eight weeks excludes the diagnosis of GBS and suggests a diagnosis of chronic inflammatory polyneuropathy (CIDP) [74]. Predictive of poorer pediatric outcome are young age, maximum disability score at presentation, quadriplegia at day 10, need for ventilation support, cranial nerves involvement, and unexcitable motor nerve conduction [5].

The overall mortality in childhood GBS is estimated to be less than 5%. Death may be caused by ventilatory failure, cardiac arrhythmias, dysautonomia and pulmonary embolism [74]. 

Although GBS is a monophasic illness, about 7% to 16% of patients suffer recurrent episodes of worsening after an initial improvement [74].

Neurophysiologic studies play a very important role in diagnosis, subtype classification, and confirmation of PNS involvement [78].

Nerve conduction studies (NCS) rely on abnormalities in motor nerves to identify features of demyelination, with sensory nerve conduction studies helping to differentiate different forms of axonal GBS, that is AMAN from AMSAN. The diagnostic yield of NCS is increased by studying at least three sensory and four motor nerves, in addition to F-waves and H-reflexes [73]. The classical findings on NCS include the presence of a partial motor conduction block, abnormal temporal dispersion of motor responses, prolonged distal motor and F-wave latencies, and reductions in maximum motor conduction velocity [72, 73]. Electromyography (EMG) shows evidence of acute denervation of muscle [78]. There is no particular best time to do nerve conduction studies, although they should be done as soon as possible after presentation and the studies should be repeated after 1 or 2 weeks if the initial studies are non-diagnostic or do not allow adequate neurophysiologic classification [78].

CSF examination is essential for GBS diagnosis. Characteristically the CSF protein is raised with <10 mononuclear cells/mm3 (albuminocytologic dissociation). In the first days of the disease the CSF may be normal [5].

The potential mortality rate (5% in children) necessitates hospitalization and regular monitoring of autonomic and respiratory function until maximal disability is reached [5].

Patients with slow progression may simply be observed for stabilization and spontaneous remission without treatment [5, 79]. 

Intravenous immunoglobulin or plasma exchange (PE) are treatment options in severe childhood GBS [80, 81]. Recent American Academy of Neurology practice guidelines recommended treatment (with PE or IVIG) for GBS patients who were considered immobile [79]. Recovery is hastened equally by these two treatment but IVIG is preferred in childhood because of less invasiveness and rarer complications than PE [82, 83]. 

Trials combining IVIG with either plasmapheresis or immunoadsorption have failed to demonstrate additional benefits when compared to treatment with IVIG alone [5]. 

Experimental evidence shows IVIG may prevent axonal degeneration in acute motor axonal neuropathy [5]. A commonly recommended protocol is IVIG 0.4g/Kg/day for 5 consecutive days. However a trial of 50 children found no significant difference in outcome when IVIG was given over 2 days rather than 5 days [84]. The efficacy of IVIG has been proved within 2 weeks of symptom onset, so that beyond this time it remains uncertain [73, 83]. Moreover there is no evidence that a second course of IVIG is beneficial, but this is often used when patients deteriorate or fail to improve 7- 10 days after a first course of IVIG [83].

PE is generally safe in children who weigh 10 kg or more. As a rule, children with sufficiently severe symptoms should receive a series of exchanges with a cumulative total of approximately 250 ml/kg volume exchange or roughly a triple-volume exchange to justify treatment [74]. Trials assessing the efficacy of PE have established that it is effective within the first 4 week of illness [73, 83].

There is no definitive role for corticosteroids in the treatment of children with GBS at present. Corticosteroids alone should not be used in GBS therapy [85]. Combined intravenous methylprednisolone and IVIG showed a minor synergistic effect however the potential importance of combination treatment with the corticosteroids and IVIG, warrants further investigation [86].****

Studies on adults show that immunoadsorption and CSF filtration are effective. CSF filtration possibly removes inflammatory mediators such as sodium channel-blocking pentapeptide. Exchange transfusion appears effective in pediatric case reports [5, 87]. However present results are not definitive to recommend the use of these treatment options [5, 88]. 

There is interest in old and newer drugs which may be promising for GBS treatment in the near future. Potential therapies include interferon beta-1a, cyclooxygenase-2 inhibitors, complement-blocking agents, sodium channel blockers (axonal protection) and trophic factors [78, 88]. Present evidence is insufficient to recommend the use of these therapies [5]. 

Inhibitors of complement activation that prevent the formation of membrane attack complex are highly effective in abrogating neuronal and neuromuscular junction damage in animal models [89].

Pain is a common feature in GBS in the acute stages. Paracetamol, nonsteroidal anti inflammatory drugs, and opiates are useful. Drugs for treatment of neuropathic pain are usually of some benefit, including gabapentin, carbamazepine, and amitriptyline [83]. 

## CHRONIC INFLAMMATORY DEMYELINATING POLYRADICULONEUROPATHY

Children with CIDP present with a subacute onset of symmetric proximal and distal weakness that progresses over at least 2 months. CIDP is closely related to Guillain Barrè syndrome and it is considered the chronic counterpart of the acute disease. Its symptoms are also similar to progressive inflammatory neuropathy [74]. The two mandatory clinical research criteria for the diagnosis of CIDP include the following: (1) progressive or relapsing motor and sensory dysfunction of more than one limb and (2) hyporeflexia or areflexia, which usually involves all four limbs [90]. 

The estimated prevalence is 0.48 per 100, 000 children with a male preponderance and antecedent illnesses or vaccinations occur in approximately half of patients [91]. Onset occurs from birth, with most childhood cases occurring before 10 years of age [5].

The clinical manifestations of CIDP at the time of referral and at a more chronic phase of the illness may be extremely variable [91]. Children present mainly with progressive weakness and loss of neurological function primarily in the legs and arms. Proximal and distal limbs are commonly affected in roughly symmetrical pattern [5]. Some patients present with a progressive sensory ataxia; in other patients motor deficits predominate [92]. Only a minority of children present with sensory symptoms such as tingling and numbness of hands and feet; however, sensory findings occur in most children [5]. Cranial nerve and respiratory muscle involvement are uncommon, although both may occur. Autonomic system dysfunction can appear; in this case, the patient would complain of orthostatic dizziness, problems with bowel and bladder functions, and cardiac problems [88]. Presentation may be monophasic with complete recovery, relapsing remitting, slow-progressive, or stepwise progressive, and should be distinguished from acute immune neuropathies and hereditary motor and sensory neuropathies [5].

The diagnosis is made clinically with the support of electrophysiological studies which show evidence of a motor and sensory demyelinating polyradiculoneuropathy, often patchy and often with evidence of conduction block or temporal dispersion that distinguish CIDP from hereditary demyelinating neuropathies [89]. The diagnosis is supported by the presence of raised CSF protein without cells, thickened and/or enhancing roots on MRI of the cervical or lumbar spine, a positive response to immunomodulatory treatment or unequivocal quantitative biopsy features consistent with demyelination and remyelination with or without actively visualized macrophage-associated demyelination [6, 89].

There have been no randomized trials regarding first-line treatment in pediatric patients, which is in part related to the rarity of this disease. Therefore, treatment for pediatric patients has paralleled the treatment used in adults, included intravenous immunoglobulin, corticosteroids, PE, or other immunosuppressive medications [93]. When these treatments were compared no significant difference in efficacy was found, so the initial treatment is often chosen on the basis of its cost, availability, and side effects [89, 94].

Steroids induced short-term improvement in 71-100% of children in small series. The best regimen in children is not known. Initially 1-2 mg/kg/day oral prednisone (maximum 60-80mg) usually results in improvement within 1- 4 weeks. Slow tapering is begun after significant improvement, but not before 4-6 weeks. Relapses are frequent with rapid tapering; therefore, a slow reduction by 5mg every 2 weeks is recommended after a clinical response. Deterioration may occur within days of starting steroids. Intermittent pulse intravenous methylprednisolone may have less corticosteroid side effects over the longer-term than oral corticosteroids [5]. Adverse effects of prednisone seen in pediatric patients include increased appetite, emotional lability, weight gain, cushingnoid features, hypertension, and growth retardation. Other adverse effects are well known to occur with prolonged corticosteroid treatment, including increased susceptibility to infection, increased blood glucose, osteoporosis, development of cataracts. Children treated chronically with corticosteroids may have persistent weight gain even after the cessation of therapy [95].

IVIG (at a dose of 0.4 mg/kg/day for 5 consecutive days) have showed good efficacy in 50-88% of pediatric CIDP cases, improving disability for 2-12 weeks and may be used as initial therapy because of paucity of side effects. A proportion of patients need recurrent treatments (months-years) to maintain improvement [96]. 

Adverse effects of IVIG in pediatric patients are mild and without long-term consequences. They include headaches, myalgias, and malaise. They can be mitigated with pretreatment and treatment during the infusion with analgesics, nonsteroidal anti-inflammatory drugs, and antihistamines, as well as by maintaining adequate hydration and decreasing the infusion rat [95]. 

A new development is the use of subcutaneous immunoglobulin (ScIg) but its efficacy has not been tested in pediatric patients. Two adult CIDP patients treated with ScIg have been reported. Application of ScIg was well tolerated, easy to manage, and led to stabilization of the disease course [96].

PE showed good short-term improvement in 50-100% of children in small CIDP series [5]. Plasmapheresis is less often used in children because it requires the installation of central catheters, with potential infectious and thrombotic complications, as well as hemodynamic complications of the treatment itself [93]. Two to three exchanges for week may be given for 6-10 treatments or until improvement, and then frequency tapered over several months. PE should be reduced gradually and occasionally used with immunosuppressive due to possible rebound worsening. Patients refractory to immunosuppressive/IVIG may improve when PE is followed immediately by IVIG [5]. 

Only a minority of studies report treatment outcomes with immune-modulating drugs in childhood chronic inflammatory demyelinating polyneuropathy. Treatments used include azathioprine, methotrexate, cyclosporine and cyclophosphamide [93]. 

In childhood, there is the most experience with azathioprine, although it is still limited. Some authors reported using azathioprine usually as a second-line agent when corticosteroid had failed or as a steroid-sparing agent [97]. The efficacy of azathioprine in childhood is still unclear. A favorable response had been reported in some patients while, in other series of childhood CIDP patients, azathioprine therapy failed [98, 99]. Side effects include nausea, vomiting, diarrhea, rash, leucopenia, altered liver function, infection and risk of neoplasia. Ten percent of the population is heterozygous and 0.3% homozygous for inability to metabolize azathioprine, and decreased serum thiopurine methyltransferase levels identify these patients [5, 89, 96, 98, 100].

Methotrexate showed good response in children refractory to prednisolone, IVIG, azathioprine and cyclosporine. Side effects are hematological, nephrotoxic, hepatic, gastrointestinal, pulmonary and ocular [5, 99]. Cyclosporine and other calcineurin inhibitors, including tacrolimus, block the activity of the phosphatase calcineurin necessary to activation and proliferation of T-cell subsets. Cyclosporine (5mg/day) has been beneficial in some patients with CIDP. Clinical response was characterized by either decreased frequency of recurrent weakness or normalized motor function. Side effects include nephrotoxicity, electrolyte disturbances, hypertension, nausea, edema, neurotoxicity, and hirsutism [5, 101, 102].

Cyclophosphamide was used in one patient (at a dose of 4 mg/kg per day) for a period of 9 months, with improvement apparent within 1 month of onset of therapy and sustained after cessation [103]. 

Adults and childhood with CIDP have shown improvement with interferon α, β in several small studies [5, 96, 97, 101, 103]. Side effects include flulike symptoms, hypothyroidism, and hepatic toxicity [101]. In children with MS responded remarkably to INF-β, the development of CIDP has been described and one could consider the possibility that IFN treatment somehow contributed to the development of CIDP [104]. Other agents such as mycophenolate mofetil, etanercept (tumour necrosis factor alpha antagonist) and tacrolimus (FK-506) are potential treatments for CIDP but the efficacy and safety in children has not been reported [5, 105].

The future of treatment for CIDP may lie in more specific biological agents targeted at key points in the pathophysiological pathway. Rituximab is an agent on which attention is focused for future RCTs [89]. Only one paediatric report of monoclonal antibody use showed persistent relapses on Rituximab (anti- CD20) [5, 93]. Alemtuzumab (anti-CD52) achieved remission in a 19-year-old unresponsive to IVIG, prednisolone and azathioprine [106]. Side effects include hypotension, fever, chills, rash, hypogammaglobulinemia and immunesuppression [5].

## Figures and Tables

**Table 1:** Classification of the Idiopathic Inflammatory Demyelinating Disease of CNS

Idiopathic Inflammatory Demyelinating Disease of CNS
*Monofocal forms*
Acute hemorrhagic leukoencephalitis
Optic neuritis
Spinal Cord lesions:
acute necrotizing myelitis transverse myelitis
Pontine and extrapontine myelinolysis
*Multifocal forms*
Multiple sclerosis
Acute disseminated encephalomyelitis
Neuromyelitis optica or Devic’s syndrome
*Forms with sclerosis*
Baló’s concentric sclerosis
Schilder’s disease

**Table 2:** Classification of the Idiopathic Inflammatory Neuropathies

Idiopathic Inflammatory Neuropathy
*Acute*
Acute inflammatory demyelinating polyradiculoneuropathy (AIDP)
Acute motor axonal neuropathy (AMAN)
Acute motor-sensory axonal neuropathy (AMSAN)
Fisher Syndrome and other regional variants
Pharyngeal-cervical-brachial
Paraparetic
Facial palsies
Pure oculomotor
*Functional variants of Guillain-Barre´ syndrome*
Pure dysautonomia
Pure sensory Guillain-Barre´ syndrome
Ataxic Guillain-Barre´ syndrome
*Subacute*
Subacute inflammatory demyelinating polyradiculoneuropathy
*Chronic*
Chronic inflammatory demyelinating polyradiculoneuropathy
Multifocal motor neuropathy with conduction block
Chronic relapsing axonal neuropathy
Chronic ataxic sensory neuronopathy

**Table 3:** Clinical, CSF and MRI Differences Between MS, ADEM, Schilder’s Disease and Devic’s Syndrome

	MS	ADEM	Schilder’s Disease	Devic’s Syndrome
**Age**	> 10 years	< 10 years	5-14 years	20-40 years
**Gender**	M> F	M= F	M= F	F> M
**Prior flu**	variable	very frequent	_	frequent
**Encephalopathy**	rare	required	_	_
**Course**	RR, SP, PP, PR	relapsing/monophasic/ multiphasic	monophasic-not remitting/ remitting/ progressive	monophasic/ relapsing
**Topography**	optic nerve cerebellum brainstem central white matter	subcortical brainstem thalamus	centrum semiovale parieto-occipital white matter	optic nerve/spinal cord
**Relapses**	slight to moderate	moderate	severe	severe
**Brain MRI**	small lesions	large, symmetric lesions	large lesions	non specific
**Spinal cord**	< 1 segment,	_	_	> 3 segment,
**MRI**	marginal			central
**CSF cells**	< 50 lymphocytes	> 50 lymphocytes	normal	> 50 PMN
**CSF oligoclonal bands**	positive	variable	negative	negative
**NMO-IgG**	< 10%	_	_	> 70%
